# Dynamic Functional Network Connectivity Pattern of the Amygdalohippocampal Complex in Individuals With Subjective Cognitive Decline

**DOI:** 10.1002/hbm.70194

**Published:** 2025-04-14

**Authors:** Chih‐Kai Lee, Xiao‐Ya Wei, Ze‐Yi Wang, Hang Zhou, Chao‐Qun Yan, Xin‐Yuan Jiang, Guang‐Xia Shi, Xu Wang, Cun‐Zhi Liu

**Affiliations:** ^1^ International Acupuncture and Moxibustion Innovation Institute, School of Acupuncture‐Moxibustion and Tuina Beijing University of Chinese Medicine Beijing China; ^2^ Dongzhimen Hospital Beijing University of Chinese Medicine Beijing China; ^3^ School of Life Sciences Beijing University of Chinese Medicine Beijing China

**Keywords:** amygdalohippocampal complex, dynamic functional network connectivity, fMRI, independent component analysis, subjective cognitive decline

## Abstract

Subjective cognitive decline (SCD) is a potential early marker of cognitive decline and dementia. The amygdalohippocampal structure and function are closely related to cognitive decline, but few studies have investigated large‐scale amygdalohippocampal brain functional network connectivity in individuals with SCD. Here, we aim to explore how the dynamic functional network connectivity (dFNC) between the amygdalohippocampal complex and other brain networks contributes to the understanding of early cognitive decline. Independent component analysis (ICA) and dFNC analysis were applied to functional magnetic resonance imaging (fMRI) data from 66 individuals with SCD to extract the amygdalohippocampal complex and identify distinct connectivity states. Cognitive performance was assessed through a composite Z score derived from a battery of neuropsychological tests. Correlation analyses were performed to examine the associations between the dFNC patterns and cognitive performance. Three distinct dFNC states were identified, each characterized by varying levels of within‐ and inter‐network connectivity, with occurrences of 65%, 18%, and 17% respectively. Cognitive function, measured using a composite Z score, was positively correlated with amygdalohippocampal‐sensorimotor network (SM) and amygdalohippocampal‐visual network (VI) dFNC in State 2. Significant correlations were observed between the amygdalohippocampal complex and the left precentral gyrus (*r* = 0.517, FDR‐corrected *p* = 0.005), postcentral gyrus (*r* = 0.487, FDR‐corrected *p* = 0.034), and multiple visual network regions, including the lingual gyrus and lateral occipital cortex (all *P*s < 0.05, FDR‐corrected). These associations remained significant after adjusting for sex and age. These findings extend the current understanding of amygdalohippocampal dysfunction in cognitive decline and demonstrate that cognitive function is associated with distinct large‐scale amygdalohippocampal network dynamics.

## Graphical abstract

This study reveals that Subjective Cognitive Decline (SCD) is characterized by dynamic, state‐dependent connectivity changes in the amygdalohippocampal complex. These changes highlight compensatory and synchronized brain states, with implications for understanding early cognitive decline and informing potential biomarkers and intervention strategies.

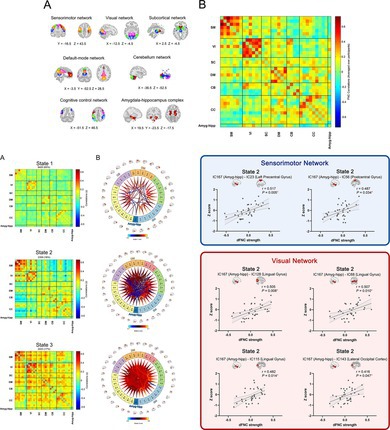

## 1 Introduction

The term subjective cognitive decline (SCD) was introduced in 2014 and has been an important clinical concept (Jessen et al. 2014a; Jessen et al. 2020). SCD refers to self‐reported complaints about memory or executive function in individuals without objective cognitive deficits on standardized assessments (Jessen et al. 2014a; Jessen et al. 2020; Rabin et al. 2017). While SCD may not always herald the onset of neurodegenerative pathology, it is increasingly recognized as a potential early marker of cognitive decline and dementia (Wang et al. 2020; Petersen 2004; McKhann et al. 2011). Therefore, understanding the fundamental brain imaging characteristics of SCD and their correlation with cognitive function is essential for early detection and intervention.

The hippocampus, a critical region in the brain for memory and cognition, has been a focal point in understanding the early cognitive changes of SCD (Caillaud et al. 2020). Structural alterations in the hippocampus, such as volumetric reduction, have been frequently associated with SCD, mild cognitive impairment (MCI), and Alzheimer's disease (AD) (Caillaud et al. 2020; Maurer and Nadel 2021; West et al. 1994), indicating that hippocampal atrophy may serve as an early indicator of aging and neurodegenerative processes (Caillaud et al. 2020; West et al. 1994; Scheef et al. 2012; van der Flier et al. 2004). The amygdala, another allocortical structure adjacent to the anterior part of the hippocampus, is also critical for the memory of emotional events (Anderson and Phelps 2001; Phelps 2006) and has been found atrophic in early AD (Poulin et al. 2011). Furthermore, combined atrophy of the hippocampus and amygdala—referred to as the amygdalohippocampal complex—has been associated with the early and accurate diagnosis of AD (Lehéricy et al. 1994; Hampel et al. 2002), and can reflect the severity of cognitive decline (Poulin et al. 2011; Hampel et al. 2002; McDonald and Mott 2017). Functionally, the amygdala‐hippocampus interaction and intracranial neuronal activity of the amygdalohippocampal area boost memory, cognitive function, and emotional responses (Hampel et al. 2002; McDonald and Mott 2017; Cahill and McGaugh 1998; Dolcos et al. 2004; Dolcos et al. 2005; Kim et al. 2013; Qasim et al. 2023). Although these findings have demonstrated the local structural and functional changes of the amygdalohippocampal complex associated with cognitive decline, relatively little is known about the spatiotemporal functional pattern of the amygdalohippocampal complex within the large‐scale brain network.

Independent component analysis (ICA) and dynamic functional network connectivity (dFNC) offer unique advantages for studying large‐scale brain functional connectivity. ICA allows for the identification of distinct neural networks by separating overlapping brain signals, providing a clearer understanding of functional connectivity patterns (Hyvärinen and Oja 2000). On the other hand, dFNC enables the examination of temporal fluctuations in brain network connectivity, shedding light on the dynamic reorganization of neural circuits underlying cognitive processes (Hutchison et al. 2013), and it is more correlated to amyloid‐β deposition rather than resting‐state static functional network connectivity (sFNC) (Hahn et al. 2019). These advanced neuroimaging techniques have the potential to reveal nuanced connectivity properties in SCD and other cognitive disorders, enhancing our understanding of the neural bases involved.

This study delves into the dFNC between the amygdalohippocampal complex and other brain networks in individuals with SCD and elucidates the intricate relationship between amygdalohippocampal dynamic connectivity and cognitive performance. We hypothesized that cognitive decline would be associated with dFNC between the amygdalohippocampal complex and cognitive‐related brain networks. Through this approach, we strive to advance the understanding of early markers of cognitive decline and highlight the potential utility of dFNC analysis of the amygdalohippocampal complex for early detection and characterization of SCD.

## 2 Methods

### 2.1 Participants

Data used in this work comprise neuropsychological tests and resting‐state fMRI scans from 66 SCD participants (56 females, mean age = 64.88 ± 5.19 years) collected at baseline in our previous clinical trial (Wang et al. 2024). The trial was approved by the Ethics Committee of the Beijing Hospital of Traditional Chinese Medicine affiliated with Capital Medical University (2017BL‐061‐02) and registered in clinicaltrials.gov (NCT03444896) (Yan et al. 2019). The identification of SCD was based on standard criteria proposed by the Subjective Cognitive Decline Initiative (SCD‐I) (Jessen et al. 2014b). The Subjective Cognitive Decline Questionnaire (SCD‐Q) was used for SCD screening (SCD‐Q score > 5) (Hao et al. 2020). Please see our previous paper for more details about inclusion and exclusion criteria (Wang et al. 2024).

### 2.2 Cognitive Function Measurement

The global cognitive function was a composite Z score derived from a battery of multidomain neuropsychological assessments (Barnes et al. 2013), including the Auditory Verbal Learning Test, Animal Fluency Test, Boston Naming Test, Trail Making Test, Stroop Color Word Test, Digit Symbol Substitution Test, Digital Span Test, and Clock Drawing Test (Wang et al. 2024). The Z scores for each test were calculated by standardizing raw scores relative to the mean and standard deviation based on the baseline assessments of the 66 participants. A composite Z score was then derived by averaging the Z scores across all domains to provide a global measure of cognitive performance. This method ensures that each cognitive domain contributes proportionally to the overall score, thereby minimizing the impact of individual test variability. Composite Z scores have been robustly and effectively utilized in prior studies to examine early and subtle cognitive changes (Wang et al. 2024; Barnes et al. 2013; Barnes et al. 2023; Valls‐Pedret et al. 2015).

### 2.3 MRI Acquisition

The MRI scan was performed with a 3.0 Tesla scanner (Skyra, Siemens, Erlangen, Germany) in the Beijing Hospital of Traditional Chinese Medicine. High‐resolution T1‐weighted brain structural MRI was obtained using a 3D magnetization‐prepared rapid gradient echo sequence: repetition time (TR)/echo time (TE) = 2530/2.98 ms, flip angle = 7°, inversion time = 1100 ms, slice number = 192, matrix = 256 × 256, voxel size = 1.0 × 1.0 × 1.0 mm, slice gap = 0 mm. Resting‐state fMRI (rs‐fMRI) was performed using an echo planar imaging sequence: TR = 2000 ms, TE = 30 ms, bandwidth = 2368 Hz/Px, echo spacing = 0.5 ms, field of view (FOV) = 224 × 224 mm, slice gap = 0.875 mm, slice number = 32, number of volumes = 240, matrix = 64 × 64, voxel size = 3.5 × 3.5 × 3.5 mm.

### 2.4 MRI Data Preprocessing

Data was preprocessed by using a MATLAB toolbox for Data Processing & Analysis for Brain Imaging (DPABI, http://rfmri.org). The main steps included the removal of the first 10 time points, slice timing, head movement correction, spatial normalization to Montreal Neurological Institute (MNI) standard brain space with a size of 3 × 3 × 3 mm, and spatial smoothing with a full width half‐maximum (FWHM) of 6 mm.

### 2.5 Independent Component Analysis

Group‐independent component analysis (GICA) was performed on all participants' preprocessed data using the GIFT toolbox (https://trendscenter.org/software/gift/). Specifically, participant‐specific data were first reduced to 230 principal components by applying principal component analysis (PCA). Next, the simplified data for all participants were concatenated across time and decomposed into independent components (ICs) using the infomax algorithm (Allen et al. 2014). The infomax‐independent component analysis (ICA) algorithm was repeated 10 times in ICASSO (Bell and Sejnowski 1995) to ensure the stability of the estimation. In order to obtain finer “functional parcellation” (e.g., an amygdalohippocampal component (Lehéricy et al. 1994; McDonald and Mott 2017; Lin et al. 2020)), a relatively high model order or number of components (i.e., 200) was used in the present study. Spatiotemporal regression and regression reconstruction approaches were used to obtain participant‐specific spatial maps and time courses. Meaningful ICs were selected based on previous literature, templates, and experience, and defined as nodes of the large‐scale network.

After extracting the ICs, postprocessing was carried out to further remove noise in the time courses of the components: (1) detrending linear, quadratic, and cubic trends, (2) conducting multiple regression of the 6 realignment parameters and their temporal derivatives, (3) despiking, and (4) low‐pass filtering with a cutoff frequency of 0.15 Hz.

### 2.6 Dynamic Functional Network Connectivity Analysis

To understand the dynamic nature of the functional connectivity and its correlation with cognitive function in the SCD population, we further performed dFNC analysis with a sliding window approach in the temporal dFNC toolkit of GIFT software. According to previous studies, a window size in the range of 30 s to 1 min is a reasonable choice for capturing dynamic modalities (Hutchison et al. 2013; Allen et al. 2014; Damaraju et al. 2014). Here, the resting‐state data of each individual were divided into windows of 22 repetition times (44 s) in steps of 1 TR to obtain a total of *W* windows. Each window's FNC matrix was calculated as pairwise correlations between the *C* selected ICs' time courses and then concatenated into a *C* × *C* × *W* array to capture temporal fluctuations in FNC. Furthermore, the GIFT toolbox incorporated an L1 norm into the graphic LASSO framework to improve the estimation of the covariance matrices among time courses of short length. To stabilize the variance of correlation coefficients, all FNC matrices were Fisher's z‐transformed.

Then, the *k*‐means clustering algorithm with the L1 distance function was repeated 100 times on the time‐varying FNC matrices to evaluate the recurring functional connectivity patterns (states). The optimal number of clusters was estimated by conducting a cluster validity analysis (silhouette) on the exemplars of all participants. To reduce redundancy between windows and computational demands, we utilized a subset of windows consisting of local maxima in functional connectivity variance as participant exemplars. Each resulting cluster represents a functional dFNC state. Next, each participant's windowed FNC matrix was assigned to one of the identified states. The participant‐specific medians of the FNC matrices at each state were estimated as the dFNC value corresponding to each group‐level state. Finally, we performed Spearman correlation analyses between the dFNC of interest IC at each state and cognitive function. Besides, partial correlation analyses were conducted controlling for age and sex, respectively. Note that not all individuals have dFNC in all states. Therefore, we only included participants with at least one window belonging to that state in the dFNC‐cognition correlation analyses. A false discovery rate (FDR) adjustment was made for the multiple comparisons.

## 3 Results

### 3.1 Independent Component Identification and Whole‐Brain Connectivity Estimation

**FIGURE 1:**
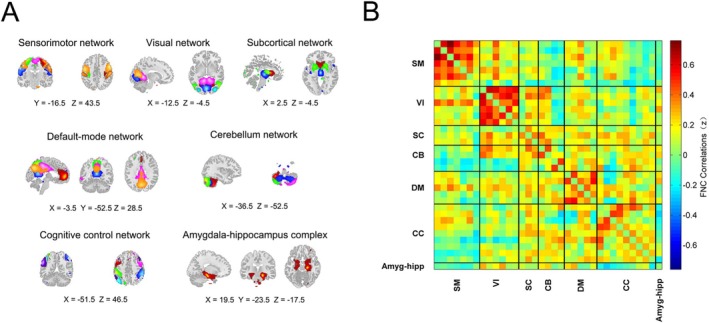
Spatial map and static functional network connectivity matrix of 7 brain networks. (A) Group‐independent component analysis identified 35 independent components, which were subsequently classified into 7 brain networks. (B) Z‐transformed group averaged resting‐state functional connectivity matrix. SM = sensorimotor network, VI = visual network, SC = subcortical network, CB = cerebellar network, DM = default‐mode network, CC = cognitive control network, Amyg‐hipp = amygdalohippocampal complex, FNC = functional network connectivity.

The 35 meaningful ICs were identified and categorized into 7 brain networks (Figure 1A), including the sensorimotor network (SM: IC12, IC13, IC23, IC56, IC100, IC161, IC191), visual network (VI: IC68, IC115, IC128, IC129, IC143, IC165), subcortical network (SC: IC16, IC33, IC124), default‐mode network (DM: IC42, IC47, IC89, IC95, IC140), cerebellar network (CB: IC43, IC52, IC65, IC77), cognitive control network (CC: IC79, IC87, IC96, IC133, IC150, IC151, IC166, IC180, IC198), and amygdalohippocampal complex (IC167). Specifically, the amygdalohippocampal complex encompasses the hippocampus and amygdala structures. Then, the 35 × 35 correlation coefficient matrices for each individual were calculated and transformed to Fisher z score matrices, resulting in sFNC.

### 3.2 Clustering Analysis and Dynamic Functional Network Connectivity

**FIGURE 2:**
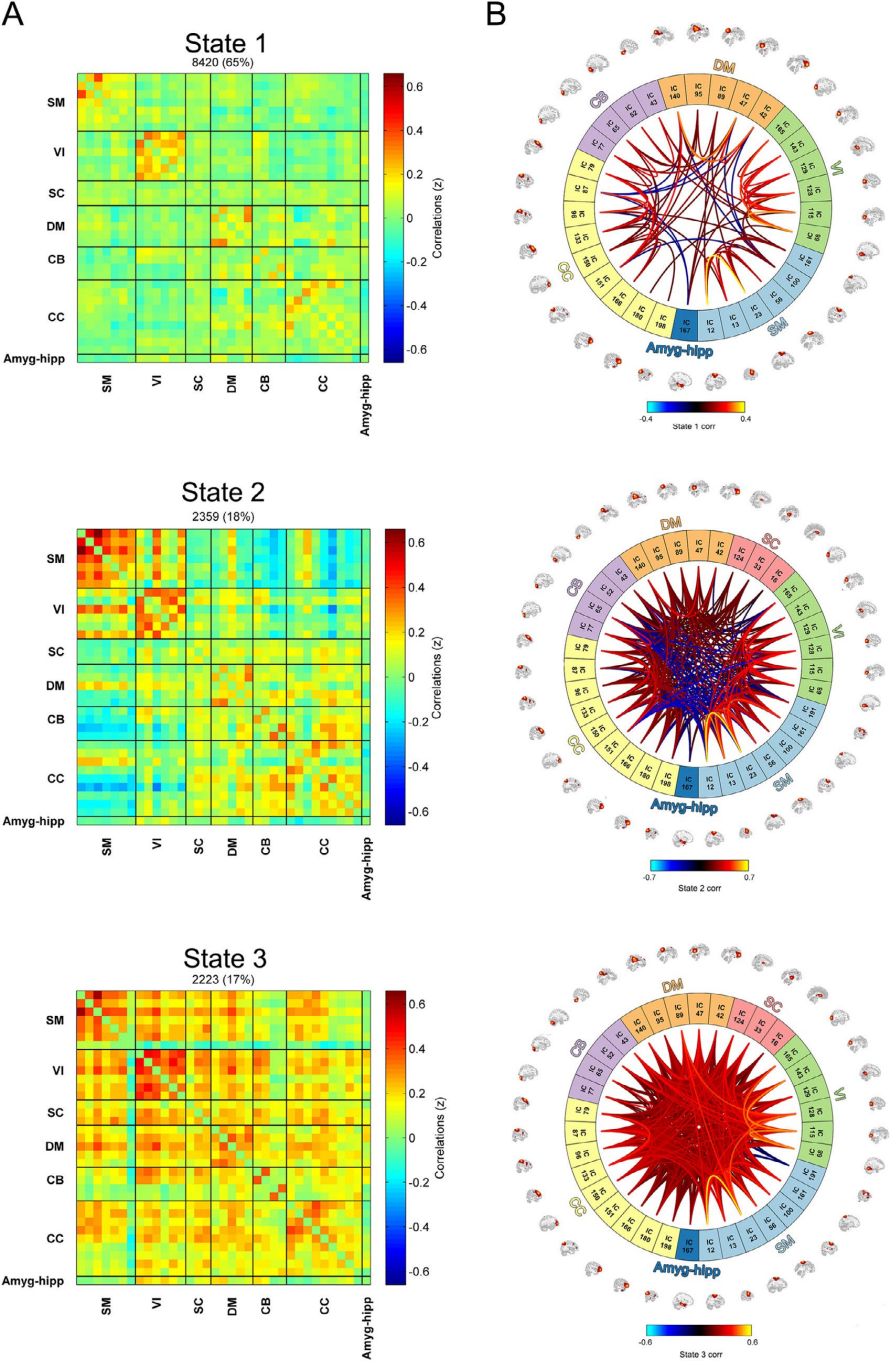
Occurrence rate and connectivity of each state. (A) Cluster centroids for each state. (B) Functional network connectivity in each state with a threshold of 0.1. SM = sensorimotor network, VI = visual network, SC = subcortical network, CB = cerebellar network, DM = default‐mode network, CC = cognitive control network, Amyg‐hipp = amygdalohippocampal complex.

The occurrence rates of 3 reliable dFNC states were determined among individuals, with State 1 manifesting at a frequency of 65%, State 2 at 18%, and State 3 at 17% (Figure 2). These states exhibited varying degrees of functional connectivity sparsity. State 1 exhibited weak connectivity strength between each network with the highest occurrence rate; in State 2, strong connectivity was observed between the SM and VI, as well as within the SM, VI, DM, CB, and CC; State 3 occurred least frequently but demonstrated the strongest overall connectivities within and between networks.

### 3.3 Cognitive Relevance of the Amygdalohippocampal Dynamic Functional Network Connectivity

**FIGURE 3:**
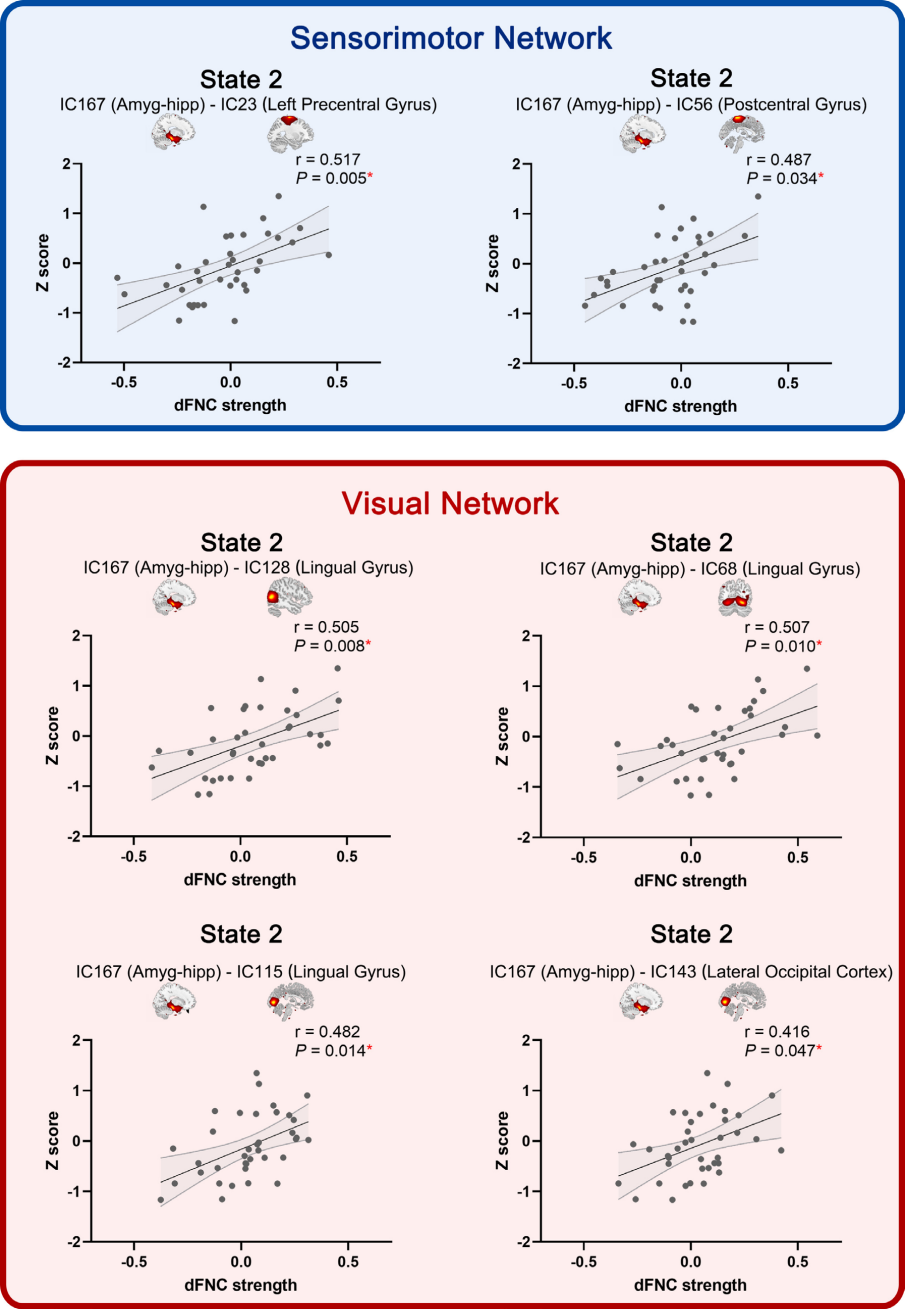
Cognitive relevance of the dFNC based on the amygdalohippocampal complex‐independent component. dFNC = dynamic functional network connectivity, Amyg‐hipp = amygdalohippocampal complex, IC = independent component. Thirty‐seven participants remained in State 2. * The correlation was significant at the 0.05 level (two‐tailed, FDR‐corrected).

We further characterized the correlation between the amygdalohippocampal dFNC with other components and cognitive function at each state (Figure 3). For each individual, the dFNC strength was the median of the windowed FNC values at each state. In State 2, there were notable positive correlations between the amygdalohippocampal‐SM (IC23, Left Precentral Gyrus; IC56, Postcentral Gyrus) as well as amygdalohippocampal‐VI (IC128, IC68, IC115, Lingual Gyrus; IC143, Lateral Occipital Cortex) dFNC and composite Z score (IC23: r = 0.517, FDR‐corrected *p* = 0.005; IC56, *r* = 0.487, FDR‐corrected *p* = 0.034; IC128: *r* = 0.505, FDR‐corrected *p* = 0.008; IC68: *r* = 0.507, FDR‐corrected *p* = 0.010; IC115, *r* = 0.482, FDR‐corrected *p* = 0.014; IC143, *r* = 0.416, FDR‐corrected *p* = 0.047). In addition, all identified dFNC‐cognition correlations remained significant when adjusting for sex or age (all *P*s < 0.05, Table 1).

**TABLE 1:** Partial correlation between dynamic functional network connectivity strength and Z score.

	Sex controlled	Age controlled
State 2 IC23	*r* = 0.550^a^ *p* < 0.001^b^	*r* = 0.517^a^ *p* = 0.001^a^
State 2 IC128	*r* = 0.524^a^ *p* = 0.001^a^	*r* = 0.505^a^ *p* = 0.002^a^
State 2 IC56	*r* = 0.476^a^ *p* = 0.003^a^	*r* = 0.487^a^ *p* = 0.003^a^
State 2 IC68	*r* = 0.515^a^ *p* = 0.001^a^	*r* = 0.507^a^ *p* = 0.002^a^
State 2 IC115	*r* = 0.484^a^ *p* = 0.003^a^	*r* = 0.482^a^ *p* = 0.003^a^
State 2 IC143	*r* = 0.449^a^ *p* = 0.006^a^	*r* = 0.416^a^ *p* = 0.012^a^

^a^ The correlation was significant at the 0.05 level (two‐tailed).

^b^ The correlation was significant at the 0.001 level (two‐tailed).

## 4 Discussion

In this study, we investigated the dynamic functional connectivity of the amygdalohippocampal complex in individuals with SCD using ICA and dFNC methods. Our results revealed three distinct connectivity states with varying patterns and sparsity, and we identified significant associations between the amygdalohippocampal complex's dynamic connectivity and cognitive performance, particularly with regions such as the precentral gyrus, postcentral gyrus, lingual gyrus, and lateral occipital cortex. These findings suggest that the dynamic connectivity of the amygdalohippocampal complex may serve as a sensitive neural marker of early cognitive changes in SCD, shedding light on its underlying pathophysiology and potential heterogeneity.

### 4.1 The Neurophysiology Basis of the Amygdalohippocampal‐Independent Component

The amygdalohippocampal complex, consisting of the amygdala and hippocampus, plays a pivotal role in emotional regulation, memory processing, and cognitive control (Caillaud et al. 2020; Maurer and Nadel 2021; Phelps 2006; Cahill and McGaugh 1998). Despite the structural independence of the amygdala and hippocampus, they exhibit functional interconnections that are pivotal for coordinating emotional responses and memory formation (Poulin et al. 2011; McDonald and Mott 2017; Qasim et al. 2023). The amygdala and hippocampus exhibit synchronized theta (4–8 Hz) and gamma (30–100 Hz) oscillations, which are essential for cognitive functions such as attention, memory encoding, and emotional regulation (Paré and Gaudreau 1996; Bauer et al. 2007). The synchronization of these oscillatory patterns enables the integration of emotional and cognitive information, thereby enhancing memory performance, especially when emotions are involved. Previous studies have demonstrated that the amygdala modulates hippocampal activity, particularly through projections that influence memory consolidation under emotional contexts (Dolcos et al. 2004; Dolcos et al. 2005; Narayanan et al. 2007). Conversely, the hippocampus sends feedback signals to the amygdala, modulating emotional responses based on memory retrieval (Roesler et al. 2021; Fanselow and Dong 2010). This bidirectional interaction underscores the amygdalohippocampal complex's integrative role in supporting both emotional and cognitive processes.

In the present study, we identified the amygdalohippocampal IC in individuals with SCD for the first time, highlighting its potential as a sensitive neural marker of early cognitive changes. This novel finding underscores the functional importance of the amygdalohippocampal complex in the SCD population and provides a foundation for further exploration of its dynamic connectivity patterns in the preclinical stages of cognitive decline.

### 4.2 Dynamic Connectivity With the Amygdalohippocampal Complex

In addition, we identified 3 reoccurring dFNC states characterized by different connectivity patterns. State 1, characterized by moderate within‐network and weak inter‐network connections, exhibited the highest proportion of occurrence, resembling the “ground state” or “static state” (Viviano et al. 2017) identified in previous research of low back pain, PD, schizophrenia, and autism spectrum disorder (Fu et al. 2018; Fiorenzato et al. 2019; Tu et al. 2020; Fu et al. 2019). This state likely represents periods of mind‐wandering or disengaged mental activity (Kucyi and Davis 2015); thus, a weaker connection of this state may reflect a stable baseline configuration of large‐scale brain networks.

State 2 was a less frequent but more complex reorganization, characterized by stronger interactions within the SM, VI, DM, CB, and CC networks, alongside weaker connectivity between SM and both CC and CB networks. This state exhibits a mixture of both strong and weak connectivity between key brain networks, suggesting a dynamic reallocation of resources to optimize cognitive function in the face of potential metabolic or structural challenges. As cognitive decline progresses, large‐scale brain networks undergo reorganization to compensate for neurodegenerative changes, particularly in early disease stages (Bagarinao et al. 2019; Hu et al. 2022; Bunzeck et al. 2024). The significant correlations observed between global cognition and amygdalohippocampal dFNC in the precentral gyrus, postcentral gyrus, lingual gyrus, and lateral occipital cortex suggest that these regions may contribute to cognitive performance in this state through their specialized roles in sensorimotor integration, spatial attention, and visual processing, respectively. The selective engagement of these networks aligns with findings that cognitive impairment is associated with altered resource allocation across networks, where visual and motor processing circuits may be increasingly recruited to support cognitive function (La Corte et al. 2016).

The precentral and postcentral gyrus, integral to the sensorimotor network, plays key roles in integrating sensory input with motor output, essential for tasks requiring attention and action coordination (Seidler et al. 2010). The lingual gyrus and occipital cortex, on the other hand, are central to visual processing and visuospatial cognition (Bonner and Price 2013; Shi et al. 2020). Their engagement in State 2 might indicate heightened reliance on sensory and visuospatial systems during early cognitive decline. This finding is consistent with studies showing that SCD and preclinical AD are associated with changes in visual and motor processing circuits, which may reflect broader shifts in neural resource allocation (Bunzeck et al. 2024). This pattern may reflect a compensatory or adaptive mechanism aimed at mitigating the impact of early neurodegenerative changes.

Importantly, these findings suggest that mechanisms in SCD share similarities with those observed in MCI and early AD, indicating a continuum of functional adaptation across disease stages. While State 2 may temporarily stabilize cognitive performance, its persistence or over‐reliance could signal the brain's struggle to cope with increasing neurodegeneration. This underscores the potential value of State 2 as a neural marker for identifying individuals at higher risk for progression from SCD to MCI or AD. Further studies integrating longitudinal designs and multimodal imaging could clarify the trajectory of compensatory reorganization and its eventual breakdown during the course of AD.

State 3 represented a highly synchronized brain state with strong global connectivity, associated with more complex cognitive operations, particularly those involving integrated emotional and executive functions. While no significant dFNC‐cognition correlation was observed in State 3 in the present study, the pattern of strong connectivity across global networks suggests that this state may be linked to enhanced cognitive flexibility and resilience. Previous studies have demonstrated that increased brain network integration, especially within the DM and executive networks, is associated with improved cognitive performance and the brain's ability to compensate for early pathological changes in conditions such as SCD, MCI, and AD (Viviano et al. 2017; Wang et al. 2022; Núñez et al. 2021). This suggests that the high level of synchronization in State 3 could reflect a neural mechanism that supports cognitive resilience, facilitating the integration of emotional and executive functions, which are critical for higher‐order cognitive processes, such as decision‐making, attention, and emotional regulation. This suggests that in State 3, the brain is able to engage a more integrated network that supports cognitive resilience and higher functional efficiency.

Overall, these findings highlight the dynamic nature of brain networks in SCD, where different states may represent different strategies that the brain uses to cope with early cognitive decline. State 1 represents the “ground state”, State 2 reflects the compensatory reorganization of neural functional resources that may temporarily stabilize cognitive performance, and State 3 reveals a more globally connected state that may support higher cognitive abilities.

## 5 Limitations and Future Directions

While this study provides valuable insights into the dynamic functional connectivity of the amygdalohippocampal complex in SCD, several limitations warrant consideration. Firstly, the use of GICA, while effective for identifying group‐level functional networks and their dynamic interactions, does not explicitly differentiate contributions from the left and right hemispheres. Given the established role of lateralization in the amygdala and hippocampus in emotional and cognitive processes (Baas et al. 2004; Yue et al. 2018), future studies should incorporate methodologies specifically designed to explore the hemispheric differences. Secondly, our findings only focused on dynamic functional connectivity; further, multimodal (PET, diffusion tensor imaging, and T1 structural MRI) imaging techniques could enhance and complement the understanding of the pathological and structural basis of present findings. Furthermore, the partial volume effects introduced by larger voxel sizes may influence the observed functional connectivity patterns. Advanced higher‐resolution imaging or ultra‐high‐field MRI may mitigate the partial volume effects. Thirdly, there are many more females than males in our cohort. Future studies with larger and sex‐balanced samples are needed to elucidate the potential effects of sex on the observed dynamic functional connectivity patterns.

SCD is increasingly recognized as an early indicator of preclinical AD (Wang et al. 2020), approximately 7% to 40% of individuals with SCD who seek medical help show biomarkers (amyloid or tau pathology) consistent with the preclinical stage of AD (Jessen et al. 2018; van Harten et al. 2013; Wolfsgruber et al. 2017). Moreover, recent studies have shown strong associations between dFNC and amyloid‐β or tau pathology in cognitively normal elderly, MCI, and AD individuals (Hahn et al. 2019; Canal‐Garcia et al. 2024). Therefore, longitudinal studies integrating dFNC and AD biomarkers (e.g., cerebral spinal fluid or PET amyloid‐β and tau) could provide a more comprehensive understanding of the functional dynamic reorganization mechanisms of SCD and its relationship to AD progression. Our findings underscore the potential of dFNC analysis as a noninvasive tool for early detection of cognitive decline and pave the way for interventions targeting specific connectivity patterns to preserve cognitive function in the SCD stage.

## 6 Conclusion

In conclusion, this study advances our understanding of the amygdalohippocampal complex's role in SCD by highlighting the importance of large‐scale dynamic functional connectivity. Our findings suggest that SCD involves complex, state‐dependent brain functional dynamics that are crucial for maintaining cognitive function. These insights lay the groundwork for future research and clinical applications, emphasizing the potential of dFNC as a biomarker for early cognitive decline and as a target for therapeutic interventions.

## Conflicts of Interest

The authors declare no conflicts of interest.

## Data Availability

The data that support the findings of this study are available from the corresponding author upon reasonable request.
